# Exploration of the *Plasmodium falciparum* Resistome and Druggable Genome Reveals New Mechanisms of Drug Resistance and Antimalarial Targets

**DOI:** 10.1177/1178636118808529

**Published:** 2018-11-27

**Authors:** Annie Cowell, Elizabeth Winzeler

**Affiliations:** 1 Division of Infectious Diseases, Department of Medicine, University of California, San Diego, San Diego, CA, USA; 2 Division of Host-Microbe Systems & Therapeutics, Department of Pediatrics, University of California, San Diego, San Diego, CA, USA

**Keywords:** drug discovery, *Plasmodium falciparum*, whole genome sequencing, antimalarial resistance

## Abstract

*Plasmodium* parasites, the causative agent of malaria infections, rapidly evolve drug resistance and escape detection by the human immune response via the incredible mutability of its genome. Understanding the genetic mechanisms by which *Plasmodium* parasites develop antimalarial resistance is essential to understanding why most drugs fail in the clinic and designing the next generation of therapies. A systematic genomic analysis of 262 *Plasmodium falciparum* clones with stable in vitro resistance to 37 diverse compounds with potent antimalarial activity was undertaken with the main goal of identifying new drug targets. Despite several challenges inherent to this method of in vitro drug resistance generation followed by whole genome sequencing, the study was able to identify a likely drug target or resistance gene for every compound for which resistant parasites could be generated. Known and novel *P falciparum* resistance mediators were discovered along with several new promising antimalarial drug targets. Surprisingly, gene amplification events contributed to one-third of the drug resistance acquisition events. The study can serve as a model for drug discovery and resistance analyses in other similar microbial pathogens amenable to in vitro culture.

**Comment on**: Cowell AN, Istvan ES, Lukens AK, et al. Mapping the malaria parasite druggable genome by using in vitro evolution and chemogenomics. *Science*. 2018;359:191-199. doi:10.1126/science.aan4472. PubMed PMID:29326268; PubMed Central PMCID:PMC5925756.

*Plasmodium* parasites, the causative agent of malaria infections, rapidly evolve drug resistance and escape detection by the human immune response via genetic changes. This is due to the incredible mutability of their genomes along with the high numbers of parasites in a person’s bloodstream during an infection. As there is no effective vaccine, antimalarial medications are of vital importance in saving lives. However, *P falciparum*, the species that causes the highest number of deaths, has developed resistance to nearly every drug in the current antimalarial arsenal. Understanding the genetic mechanisms by which malaria parasites develop antimalarial resistance is essential to understanding why many drugs fail and designing the next generation of antimalarial medications.

In the article, “Mapping the malaria parasite druggable genome by using in vitro evolution and chemogenomics,”^1^ the patterns of *P falciparum* genome evolution were comprehensively assessed by analyzing the genomes of 262 *P falciparum* clones that demonstrated stable in vitro resistance to 37 diverse compounds with antimalarial activity across different stages of the parasite’s life cycle. This study was part of the Malaria Drug Accelerator (MALDA) Project, which is done by a consortium of laboratories from academia and industry. Using the method of in vitro evolution and whole genome analysis (IVIEWGA),^2^ parasites are cultured in the presence of an antimalarial compound until stable in vitro resistance can be demonstrated (Figure 1). The parasites are then cloned and subjected to whole genome sequencing, with comparison to the sensitive parent clone to evaluate genomic changes which arose during the process of resistance acquisition.

**Figure 1.**
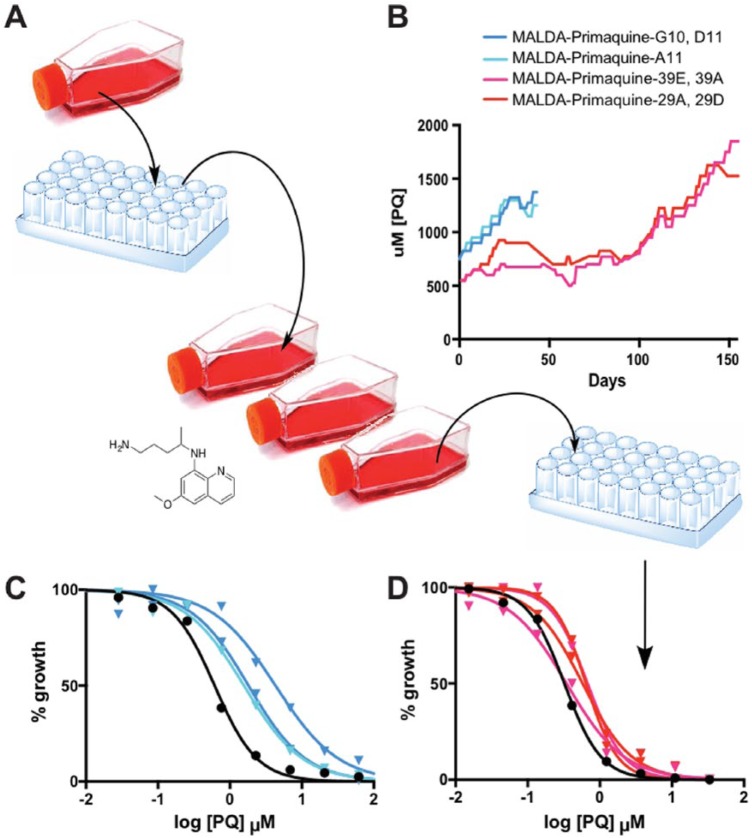
An example of generation of resistant parasites using a stepwise method of compound exposure. (A) *Plasmodium falciparum* (Dd2 strain) clones are generated using limiting dilution. Three independent clones were cultured in separate flasks in the presence of increasing concentrations of primaquine and cloned again prior to whole genome sequencing analysis. (B) The flasks of *P falciparum* were exposed to increasing concentrations of primaquine for 45 or 150 days. EC_50_ curves for the *P falciparum* primaquine-resistant clones in (C) the 45-day exposure group (MALDA-Primaquine-G10, D11, and A11) and (D) the 150-day exposure group (MALDA-Primaquine-39E, 39A, 29A, and 29D) demonstrate an increase in the EC_50_ value in the exposed clones (triangles, colored lines) compared with the sensitive parent *P falciparum* clone (circles, black lines).

## Identifying New Drug Targets for Malaria Elimination

One major goal of the study was to use probe-like and drug-like compounds with potent antimalarial activity to identify new drug targets. Such chemically validated targets are more attractive than genetically validated targets for a variety of reasons. First, they are already known to be “druggable” and often have pockets that can accommodate a small molecule with drug-like characteristics. In addition, as the small-molecule inhibitor can kill the parasite, one thus knows that the target is likely to be critically essential. Chemical inhibitors may not be able to achieve a full knock-down of activity and thus if the parasite is still debilitated in the presence of the compound, it is likely that the “target” is very important to the organism. Previous similar studies of *P falciparum* have only focused on known antimalarials or identifying a single drug target or resistance gene. Several of the compound target pairs that were discovered included dihydrofolate reductase-thymidylate synthase (encoded by *pfdhfr-ts*; PF3D7_0417200) and the benzoquinazolinone MMV027634. Although dihydrofolate reductase is a target of established antimalarials, mutations in the thymidylate synthase region of the enzyme were identified, suggesting that this portion of the protein could also be a plausible drug target. Other potential drug targets detected included farnesyltransferase (*pfftb*; PF3D7_1147500), dipeptidyl aminopeptidase 1 (DPAP1) (*pfdpap1*; PF3D7_1116700), and a predicted aminophospholipid-transporting P-type ATPase (*pfatpase2*; PF3D7_1219600).

## Identifying Drug resistance genes

The study was also important because it revealed new mediators of drug resistance. In addition to new alleles in genes known to be *P falciparum* resistance mediators, new drug resistance genes were discovered including a putative ABC transporter and a predicted amino acid transporter. Genes with premature stop codons whose products likely assist in compound detoxification were identified. This included 2 genes encoding lysophospholipases: a prodrug activation and resistance esterase and a gene which bears a FabD/lysophospholipase-like domain. It is likely that the newly identified resistance mechanisms found in this study are also important in clinical drug resistance since known antimalarial resistance mediators were repeatedly rediscovered. In fact, several potential multidrug resistance mediators identified, including the ABC transporter encoded by *pfabcI3* and the putative amino acid transporter encoded by *pfaat2*, had a nonsynonymous/synonymous mutation ratio of >2:1 in 3427 *P falciparum* clinical isolates,^3^ suggesting that the gene is under positive selection in the field parasite population. More work needs to be done on *P falciparum* isolates from the field to determine whether mutations found in this study play a role in clinical resistance to known antimalarials.

## Genome Evolution

**Figure 2.**
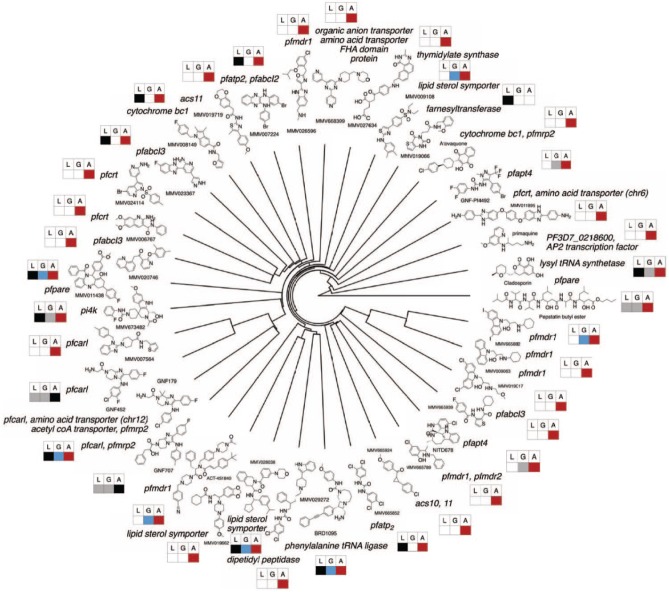
The *Plasmodium falciparum* resistome in response to 37 compounds with antimalarial activity across the parasite life cycle. The phylogenetic tree was generated based compound substructure similarity. Genes harboring mutations in independently created compound-resistant *P falciparum* clones are listed in italics for each compound. The boxes are colored to display the stage of the parasite life cycle where each compound is active. L = liver stage, G = stage 5 gametocytes, A = asexual blood stage. Boxes are gray where data are not available. EC50 cutoffs to determine whether a compound is active were ⩽1.25 µM for the asexual blood stage, ⩽1 µM for the liver stage, and ⩽2 µM for gametocytes.

The study was notable for other reasons. It was one of the most comprehensive studies published to date on how genomes evolve in the presence of drug pressure (Figure 2). In addition to nonsynonymous single-nucleotide variants (SNVs), the research revealed that copy number variants (CNVs) emerged frequently. In fact, CNVs contributed to one-third of drug resistance acquisition events. The CNVs can emerge during in vitro culture of the parasites, for example, an amplification of the *Rh1* gene has been seen in laboratory isolates but not field samples.^4^ However, gene amplifications have been associated with antimalarial resistance, including anti-folate resistance^5^ and mefloquine resistance.^6^ The high frequency with which CNVs surrounded drug targets or resistance genes further demonstrates their importance as a mediator of resistance. Large CNVs might not be stable in a clinical setting but it is difficult to determine how extensive they may be field isolates, as they are difficult to detect using whole genome amplification methods.

Another feature that was notable was that no intergenic mutations were detected that conferred resistance. It is not clear whether this was because of lower sequencing coverage in the intergenic regions or because they generally do not play as much of a role. It is logical to predict that mutations in promoters that increase transcription of a target or a 3ʹ UTR mutation that stabilizes RNA could also contribute to resistance; however, none of these events were detected with any confidence in the data set. Perhaps, better annotation of the *P falciparum* genome might improve the situation.

## Challenges and Future Directions

Although phenotypic screening of antimalarial compounds is a powerful method to identify new drugs, the study demonstrates challenges that remain in identifying the target of compounds with particular characteristics using this method of IVIEWGA. Only compounds for which resistance can be generated for *P falciparum* in vitro can be evaluated with this method. In a prior analysis of 48 antimalarial compounds, *P falciparum* resistance could not be generated to 24 compounds using this method.^7^ Developing resistant parasites was less successful with compounds that had a fast-killing rate compared with compounds with moderate- or slow-killing rates. These “irresistible” compounds could in fact be quite promising novel antimalarial compounds, as their drug targets may involve several genes which may have minimal mutational capabilities. In addition, their fast-killing rate suggests that they could lead to rapid parasite clearance in a human subject resulting in resolving of symptoms faster and appealing characteristic for the clinic.

In addition, this method of drug discovery can only be used in organisms that can be cultured readily in vitro. Although *P falciparum* causes the highest mortality worldwide, *Plasmodium vivax* is the major cause of malaria outside of Africa and leads to enormous morbidity.^8^ There are also additional challenges of controlling *P vivax* due to its ability to cause a dormant liver stage, which does not occur with *P falciparum*. However, as in vitro culture of *P vivax* is more challenging than *P falciparum*, requiring earlier-stage nucleated red blood cells which are hard to maintain, large-scale phenotypic screens are not currently possible. In addition, although there is a scalable method for phenotypic drug screening against the temporary liver stage of *P falciparum*,^9^ there is also no scalable in vitro culture system for the dormant liver stage of *P vivax* at this time, making drug screening against this important reservoir of infection challenging as well.

With the IVIEWGA method, it can also be difficult to distinguish whether a mutated gene encodes a resistance mutation or a potential drug target. For example, there were 5 different SNVs and 12 amplification events surrounding a lipid-sterol symporter (*pflss*; PF3D7_0107500), a member of the resistance-nodulation-division (RND) transporter family, in response to structurally similar compounds, suggesting a possible role as a drug target. However, RND transporters are associated with multidrug resistance in bacteria^10^ and it is currently unclear what role they mediate in *P falciparum*. Further work needs to be done to further characterize the mechanism of this lipid-sterol symporter. In addition, there are many mutations that can arise during long-term adaptation to in vitro culture. Mutations in the gene class encoding the apicomplexan AP2 transcription factor family were detected in response to a variety of different compounds. Although these could mediate *P falciparum*’s transcriptional response to drugs, this class of genes is known to regulate a variety of development transitions in the parasite^11,12^ and mediates the cellular response to stress in plants. Thus, these mutations may reflect the parasite’s response to long-term in vitro culture rather than multidrug resistance.

However, the presence of hundreds of predicted kinases, proteases, enzymes, and transporters in the 5200-gene *P falciparum* genome suggests that many potential valuable targets remain undiscovered.^13^ The data set was remarkable for having a high enrichment of mutations under positive selection. Although this study focused on genes for which there were at least 2 pieces of supporting evidence, there were also several genes with nonsynonymous mutations that may encode either potential drug targets or resistance mediators. Many of the genes are plausible targets of selection and were often found together with known resistance mutations. For example, ACT-451840–resistant clones had several nonsynonymous SNVs in *pfmdr1*; however, nonsynonymous SNVs were also observed in a putative replication factor C subunit and a putative lysophospholipase. It is also possible that the mutations detected in the data set may be compensating for fitness losses that result from the known resistance-conferring mutations. Genome editing with tools such as CRISPR-cas9 can be helpful for confirming particular resistance mutations; however, the multigenic nature of drug resistance can make it more challenging to validate the role that mutations in a single gene plays in resistance.

Despite the aforementioned caveats, this study emphasizes the overall success of the IVIEWGA method to better define the resistome and druggable genome of this deadly eukaryotic pathogen. *Plasmodium falciparum* is particularly amenable to this method as it has a haploid genome and is relatively easy to adapt to in vitro culture. This study can serve as a model for drug discovery and resistance studies for other microorganisms with similar characteristics and annotated genomes. A particular strength of using this method for drug discovery is that knowledge of the target or resistance mediator gene for a compound can aid in understanding the cause of recrudescence infections once it has moved forward to clinical trials. For example, during the phase 2a study for DSM265, a novel *Plasmodium* dihydroorotate dehydrogenase (DHODH) inhibitor, point mutations in the *dhodh* gene sufficient to cause DSM265 resistance, and a likely gene amplification surrounding the gene were identified in patients with recurrent infections.^14^ This emphasizes the importance of using DSM265 in combination therapy with another antimalarial drug to minimize the development of resistance.
